# Effects of Traditional Chinese Medication-Based Bioactive Compounds on Cellular and Molecular Mechanisms of Oxidative Stress

**DOI:** 10.1155/2021/3617498

**Published:** 2021-05-14

**Authors:** Bo Liang, Yong-Chun Zhu, Jia Lu, Ning Gu

**Affiliations:** ^1^Nanjing University of Chinese Medicine, Nanjing, China; ^2^Nanjing Hospital of Chinese Medicine Affiliated to Nanjing University of Chinese Medicine, Nanjing, China

## Abstract

The oxidative stress reaction is the imbalance between oxidation and antioxidation in the body, resulting in excessive production of oxygen free radicals in the body that cannot be removed, leading to excessive oxidation of the body, and causing damage to cells and tissues. A large number of studies have shown that oxidative stress is involved in the pathological process of many diseases, so inhibiting oxidative stress, that is, antioxidation, is of great significance for the treatment of diseases. Studies have shown that many traditional Chinese medications contain antioxidant active bioactive compounds, but the mechanisms of those compounds are different and complicated. Therefore, by summarizing the literature on antioxidant activity of traditional Chinese medication-based bioactive compounds in recent years, our review systematically elaborates the main antioxidant bioactive compounds contained in traditional Chinese medication and their mechanisms, so as to provide references for the subsequent research.

## 1. Introduction

Oxidative stress is the imbalance between oxidation and antioxidation in the body, which leads to the excessive production of oxygen free radicals that cannot be removed, resulting in excessive oxidation and thereby causing damage to cells and tissues [1–3]. Free radicals are also produced in a normal physiological state, but there are two kinds of antioxidant systems in our body: enzyme antioxidant system and nonenzymatic antioxidant system [4]. They clear free radicals produced by normal metabolism in the body to maintain the dynamic balance of free radical production and clearance and protect the body from oxidative damage. When the body is damaged exogenously or endogenously, the oxidation capacity of the body is enhanced, producing excessive free radicals and releasing a large number of reactive oxygen species (ROS). However, the reduction of antioxidant capacity makes the accumulation of excessive free radicals in the body cannot be removed, thus causing oxidative damage to the body and the occurrence of diseases [4]. Modern studies have shown that many traditional Chinese medications (TCM) and their bioactive compounds are rich in antioxidants, mainly including flavonoids, phenols, terpenes, polysaccharides, saponins, alkaloids, vitamins, and trace elements [5, 6]. Through their direct or indirect effects on the body's antioxidant system, they achieve the purpose of eliminating excessive free radicals and thus protect the body [6]. Here, we summarize these recent advances in the field of TCM-based bioactive compounds as they apply to oxidative stress. In addition, current barriers for further research are also discussed. Due to the ongoing research in this field, we believe that stronger evidence to support the application of TCM-based bioactive compounds for oxidative stress will emerge in the near future.

## 2. TCM-Based Bioactive Compounds and Oxidative Stress

### 2.1. Polyphenols

Many TCM contain polyphenols, and their antioxidant mechanism is mainly related to the hydrogen donor of their phenolic hydroxyl groups, which can bind to free radicals and terminate the chain reaction of free radicals [7–9].

As natural polyphenolic phytochemicals that exist primarily in tea, tea polyphenols have been shown to have many clinical applications [10, 11]. Tea polyphenols could protect tri-ortho-cresyl phosphate-induced ovarian damage via inhibiting oxidative stress [12] and ameliorate hepatic oxidative stress though reducing hepatic inflammation and NLRP3 inflammasome activation caused by a moderate dose of perfluorodecanoic acid [13]. Additionally, it can not only regulate the antioxidant enzyme system in the body and play an efficient scavenging effect on free radicals by activating the Nrf2/Keap1 pathway [9, 14] but also inhibit the oxidase system in the body, such as inhibiting the production of NADPH oxidase, to reduce the production of ROS in vascular endothelium and protect the heart [9, 15]. Additionally, tea polyphenols could protect PC12 cells against methamphetamine-induced reactive oxide species production through increasing the antioxidant capacities and expressions of the phosphorylation of ataxia telangiectasia mutant and checkpoint kinase 2 [16]. Tea polyphenols decrease intracellular reactive oxygen species accumulation via activating NFE2L2 and MAPK pathways in bovine mammary epithelial cells exposed to hydrogen peroxides [17]. Fresh tea leaf is unusually rich in polyphenols known as catechins which may constitute up to 30% of the dry leaf weight [18]. Catechins are ROS scavengers and metal ion chelators, whereas their indirect antioxidant activities comprise induction of antioxidant enzymes, inhibition of prooxidant enzymes, and production of the phase II detoxification enzymes and antioxidant enzymes [19]. In a paralleled, crossover, and randomized controlled study, single-dose consumption of green tea catechins influences oxidative stress biomarkers, which could be beneficial for oxidative metabolism at rest and during exercise, possibly through the catechol-O-methyltransferase mechanism [20].

Salvianolic acid is another activity of phenolic acids. Salvianolic acid A/B/C are bioactive polyphenols extracted from *Radix Salviae* (Danshen), which possesses a variety of pharmacological activities. Salvianolic acid A effectively protects the kidney against oxidative stress in 5/6 nephrectomized rats by activating the Akt/GSK-3*β*/Nrf2 signaling pathway and inhibiting the NF-*κ*B signaling pathway [21]. Salvianolic acid A ameliorates oxidation in ischemia-reperfusion-induced injury, and these protective effects may partially occur via activation of Nrf2/HO-1 and Akt/mTORC1 signaling pathways [22, 23]. Salvianolic acid A prevents Ang II-induced oxidative stress by inhibiting the activation of the Akt pathway in the macrophages [24]. Salvianolic acid B abolishes oxidative stress in the hippocampus by inhibiting NLRP3 inflammasome activation [25]. Salvianolic acid B protects the endothelial cells against oxidative stress injury by inhibiting endothelial permeability and MAPK and NF-*κ*B signaling pathways [26]. Salvianolic acid B relieves oxidative stress via inhibiting the transforming growth factor-*β*1 pathway in lipopolysaccharide-induced acute lung injury rats [27]. Salvianolic acid B protects against subarachnoid hemorrhage-triggered oxidative damage by upregulating the Nrf2 antioxidant signaling pathway, which may be modulated by SIRT1 activation [28]. Furthermore, salvianolic acid C protects the hepatocytes from acetaminophen-induced oxidative stress damage by mitigating mitochondrial oxidative stress through inhibition of the Keap1/Nrf2/HO-1 signaling axis [29]. Salvianolic acid C effectively attenuates lipopolysaccharide-induced oxidative stress via the TLR4/NF-*κ*B pathway [30].

The antioxidative effect of resveratrol *in vivo* is not to scavenge ROC directly but to play a role as a gene regulator [31–33]. Resveratrol inhibits NADPH oxidase-mediated production of ROS by downregulating the expression and activity of the oxidase [31]. Resveratrol can activate SIRT1 [34]. Studies have shown that among the established SIRT1 targets, FoxO transcription factors contribute to the antioxidative effects of resveratrol by upregulating antioxidative enzymes and eNOS [31, 34, 35]. SIRT1 inhibits the production of ROS in mitochondria through proliferator-activated receptor-coactivator-1*α* deacetylation and nitric oxide-dependent mechanism [36]. Resveratrol results in relieving oxidative stress, which may be largely associated with the alleviation of metabolic disturbances [37]. In addition, resveratrol upregulated the activities of some antioxidant enzymes by activating Nrf2 [31, 38]. Resveratrol also has effects on nonenzymatic antioxidants [31]. For example, resveratrol can upregulate *γ*-glutamylcysteine synthetase by activating Nrf2 [38], thus increasing the content of glutathione in endothelial cells [39].

Polyphenols include also flavonoids, which are a series of compounds with C6-C3-C6 as the basic carbon frame [40, 41]. Their antioxidant and anti-inflammatory activities are mainly due to their ability to prevent or inhibit reactions related to oxygen free radicals, mediate or increase the activity of antioxidant enzymes, and thus scavenging ROS [2]. They can improve the antioxidant status by weakening the activity of the NF-*κ*B pathway and inhibiting the expression of a variety of inflammatory cytokines and chemokines, such as monocyte chemoattractant protein-1, nitric oxide synthase, cyclooxygenase, lipoxygenase, cell adhesion molecules, tumor necrosis factor, and interleukin [2, 42].

Baicalein, a widely distributed natural flavonoid [43], downregulates protein kinase R-like ER kinase and upregulates Nrf2 to significantly alleviate oxidative stress [44–47]. Moreover, baicalein exerts a protective effect under oxidative stress through regulating the KLF4/MARCH5/Drp1 pathway [48, 49], stabilizing carboxyl terminus of Hsc70-interacting protein activity to promote receptor-interacting serine/threonine kinase 1/3 ubiquitination and degradation [50], and regulating PARP-1/AIF [51] and NF-*κ*B pathways [47, 52].

Baicalin, also extracted from *Scutellariae Radix* (Huangqin) [53, 54], alleviates intestinal oxidative damage by inhibiting NF-*κ*B and increasing mTOR signaling to modulate downstream oxidative responses after deoxynivalenol challenge [55, 56]. Baicalin inactivates succinate dehydrogenase to suppress ROS production and protects glutamine synthetase protein stability against oxidative stress [57]. Baicalin treatment inhibits the NF-*κ*B and p38 MAPK signaling pathways, thereby achieving its antioxidant effect in a dose-dependent manner in atherosclerosis [58]. Baicalin also protects against LPS-induced injury by decreasing oxidative stress [59] and represses C/EBP*β* via redox homeostasis [60].

Luteolin is a common flavonoid that is abundantly present in various edible plants; it is known to exhibit beneficial effects [61]. Luteolin effectively alleviates oxidative stress injury induced by hydrogen peroxide through P38 MAPK/NF-*κ*B activation [42, 62–64]. Luteolin activates the Nrf2 pathway and increases the antioxidant defense capacities of ochratoxin A-treated cells [65]. Luteolin exhibits antioxidative property in lipopolysaccharide-stimulated murine macrophages through transforming growth factor beta-activated kinase 1 TAK1 inhibition and Nrf2 activation [66]. Luteolin also enhances the antioxidative process in intracerebral hemorrhage and testicular injury by activating the p62-Keap1-Nrf2 [67] or Nrf2/HO-1 pathway [68]. Moreover, p21 upregulation and mTOR signaling inhibition are involved in the antioxidant effect of luteolin [69].

The antioxidation of quercetin is mainly the result of the joint action of the catechol group on the B ring and the free hydroxyl group (OH-) on the A ring [70, 71]. In addition, quercetin has a 3-OH group, which is an effective inhibitor of lipid oxidation and can effectively reduce the abnormal production of ROS [42, 70]. Quercetin attenuates d-galactose-induced aging-related oxidative alterations through NF-*κ*B [72], reverses lipopolysaccharide or 1,2-dimethylhydrazine-mediated oxidative stress via targeting the MAPK/Nrf2/Keap1 signaling pathway [73, 74], and improves d-galactosamine-induced cellular damage by inhibiting oxidative stress via inhibiting HMGB1 [75] and SIRT1/ER stress [76].

Silymarin can increase the activity of antioxidant enzymes, such as superoxide dismutase and catalase, so it can scavenge free radicals efficiently. Silymarin can also inhibit lipid peroxidation, so it can protect the integrity of the structure and function of hepatocytes from various oxidative damage [77, 78].

Puerarin prominently alleviated oxidative stress through TLR4/NLRP3 inflammasome activation [79], Nrf2 pathway [80, 81], and antioxidant enzymes [80] by significantly downregulating HIF-1*α* and upregulating TIMP-3 and BCL-2 [82]. Moreover, puerarin may inhibit MAPK and active STAT3 to enhance the antioxidant capacity [83].

### 2.2. Saponins

The main saponins in TCM are steroidal saponins and triterpenoid saponins. The contents of steroidal saponins were more in *Anemarrhenae Rhizoma* (Zhimu), *Asparagi Radix* (Tiandong), *Ophiopogonis Radix* (Maidong), and *Paris polyphylla* (Chonglou), and the contents of triterpenoid saponins in *Panax ginseng C.A. Mey* (Renshen), *Acanthopanax senticosus* (*Rupr. Maxim.*) *Harms* (Ciwujia), and *Cimicifugae Rhizoma* (Shengma) were higher.

The levels of malondialdehyde and lactate dehydrogenase can be reduced by timosaponin, which improves superoxide dismutase and nitric oxide [84]. The research showed that timosaponin could protect PC12 cells by reducing the level of ROS induced by hydrogen peroxide [85]. Timosaponin may have the effect of protecting INS-1 pancreatic *β* cells through reducing IL-1*β* production by inhibiting the NLRP3 inflammasome in macrophages and restoring the insulin secretion ability and cell viability by reducing oxidative stress [86]. Timosaponin can also reduce the activity of NF-*κ*B to inhibit the production of inflammatory factors and reduce the inflammatory response [84].

Ginsenoside, a potential treatment candidate for the attenuation of aging-related disease [87], produces antidepressant-like effects on chronic unpredictable mild stress-exposed rats involving protection against oxidative stress and thus the neuronal deterioration resulting from inflammatory responses [88]. Ginsenoside not only upregulates GPX4 to reduce oxidative stress and thereby alleviates 6-hydroxydopamine-induced neuronal damage [89] but also effectively attenuates D-galactose-induced oxidative stress via restoring the upstream PI3K/AKT signaling pathway [90]. Besides, ginsenoside significantly ameliorates oxidative stress through regulating SIRT1 [91]. In cardiomyocytes, ginsenoside decreases oxidative stress via activating the antioxidant signal pathway of AMPK [92, 93], PERK/Nrf2/HMOX1 [94], and Nrf2 pathways [95, 96].

### 2.3. Polysaccharides

Polysaccharides are a kind of compound composed of more than 10 glycosyl groups bound by glycosidic bonds, which is one of the four basic substances of life [97]. Polysaccharides have the characteristic of antioxidant stress. Several antioxidant mechanisms of polysaccharides include direct scavenging of ROS, enhancement of antioxidant enzyme activity, and binding of polysaccharide molecules with metal ions necessary for ROS to inhibit the production of free radicals [98–100].

Astragalus polysaccharides extracted from the dried rhizome of *Astragalus membranaceus* (Huangqi) can improve the activity of antioxidant enzymes and reduce oxidative stress indices [97, 101–103]; it alleviates hydrogen peroxide-triggered oxidative injury via elevating the expression of KLF2 via the MEK/ERK pathway [104] and alleviates tilmicosin-induced toxicity by inhibiting oxidative damage and modulating the expressions of HSP70, NF-*κ*B, and Nrf2/HO-1 pathway [105]. Astragalus polysaccharides can also effectively alleviate oxidative stress-mediated osteoporosis, which may be related to its regulation of the FoxO3a/Wnt2/*β*-catenin pathway [106]. Astragalus polysaccharides combined with matrine exert a synergistic protective effect against oxidative stress, which might be associated with regulating TFF3 expression [107].

Lycium barbarum polysaccharides from *Goji berries* or *Lycium barbarum L.* (Gouqi) could protect retinal ganglion cells from CoCl_2_-induced apoptosis by reducing mitochondrial membrane potential and ROC [108]. And Lycium barbarum polysaccharides present antioxidant effects with utility [109, 110], resulting from direct reduction of ROS, restoration of endogenous antioxidant enzymes, and downregulation of p-eIF2*α*, GRP78, and CHOP [97, 101, 110, 111].

Ziziphus jujuba polysaccharides from Ziziphus jujuba Mill (Zao) contain four fractions (one neutral polysaccharide fraction named ZJPN and three acidic polysaccharide fractions named ZJPa1, ZJPa2, and ZJPa3 separately), and their superoxide anion scavenging ability is stronger than hydroxyl radicals [112]. In addition, the acidic polysaccharide fractions show outstanding chelation to ferrous ions [101].

Other polysaccharides such as Angelica polysaccharides can increase the activity of superoxide dismutase, reduce the level of malondialdehyde, and overenhance the phosphorylation of Akt/hTERT to mitigate the harm of the peroxidation of low-density lipoprotein [113, 114]. Further, Angelica polysaccharides can upregulate miR-126, which could activate the PI3K/AKT and mTOR signal pathways, to attenuate cellular oxidative response damage [115].

*Cordyceps* (Dongchongxiacao) is a genus of ascomycete fungi that has been used for TCM [116]. The polysaccharides contained in *Cordyceps* have a good ability to scavenge DPPH and ABTS free radicals [101, 117].

## 3. Conclusions

Many TCM-based bioactive compounds are rich in antioxidants and have good development prospects. However, different bioactive compounds have different targets for inhibiting oxidative stress (see Table 1), and the side effects of various bioactive compounds have not been fully studied. Therefore, we need to further explore the antioxidant mechanisms of TCM and in-depth study the side effects of related bioactive compounds to provide protection for the treatment of related diseases.

## Figures and Tables

**Table 1:** TCM-based bioactive compounds and oxidative stress.

Bioactive compounds	Cellular and molecular mechanisms	References
Polyphenols		
Tea polyphenols	Reduce inflammation and NLRP3 inflammasome activation, regulate the antioxidant enzyme system and play an efficient scavenging effect on free radicals by activating the Nrf2/Keap1 pathway, inhibit the oxidase system, increase the antioxidant capacities and expressions of p-ATM and p-Chk2, and activate NFE2L2 and MAPK pathways.	[9, 12–17]
Salvianolic acid	Regulate Akt, Keap1/Nrf2/HO-1, TLR4/NF-*κ*B, and MAPK signaling pathways, inhibit NLRP3 inflammasome activation, inhibit endothelial permeability, and inhibit transforming growth factor-*β*1 pathway.	[21–30]
Resveratrol	Inhibit NADPH oxidase-mediated production, activate SIRT1, upregulate antioxidative enzymes and eNOS, alleviate metabolic disturbances, upregulate the activities of some antioxidant enzymes by activating Nrf2, and upregulate *γ*-glutamylcysteine synthetase by activating Nrf2.	[31, 34–39]
Baicalein	Downregulate PERK and upregulate Nrf2; regulate KLF4-MARCH5-Drp1, PARP-1/AIF, and NF-*κ*B pathways; and stabilize CHIP activity to promote RIPK1/RIPK3 ubiquitination and degradation.	[44–52]
Baicalin	Inhibit NF-*κ*B and p38 MAPK signaling pathways and increase mTOR signaling, inactivate succinate dehydrogenase to suppress ROS production, and repress C/EBP*β* via redox homeostasis.	[55–60]
Luteolin	Activate P38 MAPK/NF-*κ*B, Nrf2, and p21 pathways; inhibit mTOR signaling.	[42, 62–69]
Quercetin	Attenuate oxidative alterations through NF-*κ*B and MAPK/Nrf2/Keap1 signaling pathways; inhibit HMGB1 and SIRT1/ER stress.	[72–76]
Silymarin	Increase the activity of antioxidant enzymes; inhibit lipid peroxidation.	[77, 78]
Puerarin	Alleviate oxidative stress through TLR4/NLRP3 inflammasome activation, Nrf2 pathway, and antioxidant enzymes by downregulating HIF-1*α* and upregulating TIMP-3 and BCL-2; inhibit MAPK and active STAT3.	[79–83]
Saponins		
Timosaponin	Reduce MDA and LDH, improve SOD and NO, reduce ROS, reduce IL-1*β* production by inhibiting the NLRP3 inflammasome, and reduce the activity of NF-*κ*B.	[84–86]
Ginsenoside	Upregulate GPX4; restore the PI3K/AKT signaling pathway; regulate SIRT1; and activate AMPK, PERK/Nrf2/HMOX1, and Nrf2 pathways.	[88–96]
Polysaccharides		
Astragalus polysaccharides	Improve the activity of antioxidant enzymes and reduce oxidative stress indices; alleviate oxidative injury via elevating the expression of KLF2 via the MEK/ERK pathway; inhibit oxidative damage and modulate the expressions of HSP70, NF-*κ*B, and Nrf2/HO-1 pathway; and regulate FoxO3a/Wnt2/*β*-catenin pathway.	[97, 101–106]
Lycium barbarum polysaccharides	Reduce mitochondrial membrane potential and ROC, reduce ROS, restore endogenous antioxidant enzymes, and downregulate p-eIF2*α*, GRP78, and CHOP.	[97, 101, 108–111]
Ziziphus jujuba polysaccharides	Strong superoxide anion scavenging ability; outstanding chelation to ferrous ions.	[101, 112]
Angelica polysaccharides	Increase SOD, reduce MDA, and overenhance the phosphorylation of Akt/hTERT; upregulate mir-126, which could activate the PI3K/AKT and mTOR signal pathways.	[113–115]
Cordyceps polysaccharides	Good ability of scavenging DPPH and ABTS free radicals.	[101]

## Data Availability

The data used to support the findings of this study are included within the article.
